# Percutaneous management of post-thrombotic syndrome: Early experience with a novel thrombectomy catheter designed to address chronic venous occlusions

**DOI:** 10.1016/j.jvscit.2026.102250

**Published:** 2026-04-11

**Authors:** Conor Reid, Andrew D. Brown

**Affiliations:** a Division of Vascular Interventional Radiology, Department of Medical Imaging, St Michael’s Hospital Unity Health Toronto, Toronto, ON, Canada; b Department of Medical Imaging, University of Toronto, Toronto, ON, Canada

**Keywords:** Intervention, Venous, Thrombectomy, Post-thrombotic Syndrome

## Abstract

**Background:**

Post-thrombotic syndrome (PTS) is a chronic sequela of deep vein thrombosis, often associated with significant morbidity and limited treatment options in advanced cases. The VenaCore thrombectomy catheter (Inari Medical, Irvine California) has been developed to address chronic venous occlusions without the need for stenting.

**Methods:**

This retrospective single-center review examined the first eight consecutive patients with PTS treated with the VenaCore catheter at our institution between September and December 2024. Clinical data, procedural details, technical success, safety outcomes, and 30-day follow-up imaging and symptom assessment (Villalta score) were evaluated. A comprehensive description of our endovascular approach and device technique is provided.

**Results:**

Eight patients (4 female, 4 male; median age 39 years) were treated. The median time since deep vein thrombosis diagnosis was 2 years (range, 6 months to 20 years). All patients had moderate to severe PTS symptoms. Technical success was achieved in seven of the eight patients (88%). The median length of occlusion crossed was 20 cm. A mix of both iliofemoral and femoropopliteal occlusions were treated (4:4). The median VenaCore device time was 10 minutes, with luminal gain observed in all cases. One vessel rupture was encountered (managed successfully). At the 30-day follow-up, treated segments remained patent in six of the seven successfully recanalized patients (86%). Improvement in symptoms was reported by these six patients, with a mean reduction in Villalta score of 4.8 (interquartile range, 4.0-5.0) observed.

**Conclusions:**

In our initial experience, the VenaCore thrombectomy catheter demonstrated promising technical performance in improving luminal gain and symptom relief in patients with PTS, with an acceptable safety profile. Further studies are warranted to assess long-term patency and clinical durability.

Deep vein thrombosis (DVT) is a condition that results in significant morbidity across the globe. In the United States, upwards of 900,000 people are affected by venous thromboembolism on an annual basis.^1^ In Europe, approximately 648,000 patients are impacted each year.^2^ In those patients diagnosed with DVT, 20% to 50% go on to develop post-thrombotic syndrome (PTS).^3^ PTS is a chronic condition that can result in an array of symptoms including pain, swelling, and venous claudication in the affected limb. In severe cases of PTS, skin changes ranging from discoloration to ulceration may be encountered.^4^ PTS is recognized as a major cause of long-term morbidity with a high socioeconomic burden.^5^ Despite its potential sequelae and impact on patients lives, the condition presents significant treatment challenges with limited therapeutic options.

Acute DVT has traditionally been managed with anticoagulation; however, in recent years we have seen increasing interest in the utility of endovascular therapies within this patient cohort.^6^ This has led to the development of numerous devices that focus on the removal of acute clot from the affected vein. Given the pathophysiology of PTS development, where persistent occlusion of the vein induces venous hypertension, promotes venous inflammation, and results in vein wall fibrosis, one of the main perceived benefits of clearing the vein in the acute stage of the disease is in reducing the risk of developing PTS.^7^ Although the true extent to which this risk is reduced is the subject of ongoing research, in the those patients who have developed PTS, current guidance predominantly focusses on anticoagulation and elastic compression stockings.^8^ Endovascular intervention continues to be reserved for patients with particularly troublesome symptoms who have failed conservative management.

In those patients with occlusion of the iliofemoral segment, improvement in symptoms and high patency rates have been described with stenting of the occluded segment.^9^ In situations, however, where the disease process is centered on the femoral-popliteal segment, the potential benefit of angioplasty and stenting is less clear with a scarcity of data available. Although some symptomatic improvement has been shown in a small number of studies after balloon angioplasty, long-term patency remains uncertain given that the these patients are generally unsuitable for stent placement on account of poor inflow through the popliteal vein.^10^^,^^11^ Attention has, therefore, turned to ways in which these challenging chronic occlusions may be treated without the need for stenting.

To address these difficult cases, the VenaCore thrombectomy catheter has been developed by Inari Medical. The device is designed to address challenging venous occlusions in patients suffering from the long-term complications of DVT. The device consists of a rotating coring element that can also be used to grasp, with the goal of engaging, separating, and removing challenging venous occlusions. As one of the first institutions to use the VenaCore thrombectomy catheter, this paper aimed to report on our early experience with the device.

## Methods

Consecutive patients with PTS who underwent percutaneous treatment at our institution between September 2024 and December 2024 were included in this retrospective review. All patients were treated with the VenaCore device. Patient consent for inclusion in this retrospective analysis was obtained and ethical approval was obtained from our institutional research ethics board.

### Data collection

Data were collected through a review of preprocedural and postprocedural consultation notes. Details of previous DVT were recorded, namely, date of initial diagnosis, location of thrombus, and all prior treatments. The most recent preprocedural imaging was reviewed to assess the extent of disease. Preprocedural symptoms were recorded. The Villalta score and Clinical Etiological Anatomical Pathophysiological score were calculated for each patient.

Intraprocedural details were examined through a review of imaging and procedural reports. Data recorded included access site details, length of the occlusion on venography, crossing devices used, time required to cross the occlusion, and overall procedural time. Time using the VenaCore thrombectomy catheter was recorded as well as volume of thrombus removed. The associated use of other devices in attempted recanalization was also recorded, namely balloon angioplasty and the ClotTriever device. All patients were followed up with a clinic consultation 30 to 60 days post procedure to assess for interval change in symptoms and procedure related complications. Postprocedural ultrasound (US) examinations were performed to assess patency of the treated venous segment in the early postprocedural period, days 30 to 60.

The primary end point was symptomatic improvement at initial follow-up review. Technical success was considered as successful recanalization of the occluded segment, and the secondary end point of vessel patency at initial postprocedural US was evaluated.

### Endovascular procedure

All procedures were performed by a single interventional radiologist with subspecialist expertise in the management of venous disease. The procedure was performed using local anesthetic and conscious sedation (fentanyl and midazolam) in all patients. Patients were positioned supine on the fluoroscopy table for the duration of the procedure. Access was obtained from a variety of access points and using a variety of access sheaths (Table I). In cases where popliteal access was required, access was obtained while the patient was positioned prone on the stretcher before transferring patient to the fluoroscopy table.

**Table I:** Procedural details

Patient	Site of disease	Length of occlusion	Access	Crossing catheter	Time to cross	VenaCore time	Angioplasty/other devices
1	Right femoral-popliteal vein	12 cm	Right common femoral vein-7F sheath upsized to 13F (VC from CFV)	Kumpe catheter and stiff glide, subsequently 0.35 Navicross, 0.18 Navicross, and V18 wire	20 minutes	10 minutes	Angioplasty pre and post VC. ClotTriever used post VC.
			left anterior tibial vein-5F radial sheath				IVUS used.
2	Left common femoral-superficial femoral vein	6 cm	Right internal jugular vein-7F sheath upsized to 24F Protrieve.	Triforce crossing system and stiff glide wire	6 minutes	5 minutes	Angioplasty pre and post VC.
			Left popliteal vein-micropuncture upsized to 13F (VC from popliteal)				ClotTriever used post VC.
3	Left femoral vein-popliteal-tibial vein	30 cm	Left common femoral vein-7F sheath upsized to 13F (VC from CFV)	0.35 Navicross and stiff glide wire followed by 0.18 Navicross, and V18 wire	25 minutes	7 minutes (femoral segment)	Angioplasty before and after VC.
			Left anterior tibial vein-4F radial sheath				ClotTriever used after VC.
4	Left external iliac and femoral vein	15 cm	Right internal jugular vein-7F sheath upsized to 20F Protrieve	MPA catheter and stiff glidewire	10 minutes	10 minutes	Angioplasty post Venacore. ClotTriever after VC.
			Left popliteal vein-7F				
5	Left common iliac - external iliac - common femoral - superficial femoral vein	10 cm	Right internal jugular vein-7F upsized to 24F Protrieve	Triforce and stiff glidewire	20 minutes	8 minutes	Angioplasty before and after VC.
			Left short saphenous vein-micropuncture upsized to 13F				
6	Left external iliac vein-common femoral vein	10 cm	Right internal jugular vein-7F upsized to 24F Protrieve	Triforce and stiff glidewire	20 minutes	6 minutes	Angioplasty before and after VC.
			Left popliteal vein-micropuncture upsized to 13F				
7	Left common-external iliac vein	22 cm	Right internal jugular vein-7F upsized to 20F Protrieve	Triforce and stiff glidewire	15 minutes	9 minutes	Angioplasty before. Rupture with VC. Balloon tamponade post.
8	Right femoral vein-popliteal vein	20 cm	Right common femoral vein-7F upsized to 13F	0.18 Navicross and V18	24 minutes	12 minutes	Angioplasty pre and post VC.
			Right posterior tibial vein-5F radial sheath				

*CFV*, common femoral vein; *VC,* VenaCore.

The objective in all cases was to obtain access both above and below the lesion, thereby providing the option to cross in either direction. Initial access was obtained with standard vascular sheaths, generally a 7F sheath at either the jugular, femoral, or popliteal vein and a 4F to 5F radial sheath if pedal access was required via the anterior or posterior tibial veins. A variety of catheter and wire combinations were used to cross the occluded segments (Table I). After successfully crossing the lesion, upsizing was performed to a 24F ProTrieve sheath (right internal jugular vein) or 13F ClotTriever sheath (femoral/popliteal) (Inari Medical). Through-and-through stiff wire access was also obtained to improve wire stability before device use. In all cases, pulmonary embolic protection was used, using the in-built mesh funnels on both the ProTrieve and ClotTriever sheaths. Intravascular US (IVUS) examination was used in our first case, but the remaining cases were performed solely using angiographic imaging. Predilatation with balloon angioplasty was performed along the length of the occluded segments before deployment of the thrombectomy catheter. This was performed to expand the channel and facilitate passage of the device. Post-thrombectomy angioplasty was used to expand and consolidate the newly recanalized segment. A variety of 0.35 Mustang balloons were used (diameter range, 5-12 mm) (Boston Scientific). Sizing was based on site of the occlusion and angiographic appearances of the patent segment both cranial and caudal to the occluded segment.

### Device technique

The VenaCore catheter has a 12F outer diameter and an effective length of 80 cm. It consists of a retractable nitinol coring element which has a variable diameter ranging from 6 to 16 mm. The coring element is contained within an outer coaxial shaft and is exposed by advancing the element out of this shaft when in a satisfactory position. The diameter can then be altered using a dial on the device handle. The coring element can also be contracted/expanded using a lever on the device handle, allowing for grasping of intraluminal material. The coring element is manually rotated in both directions through 360° by rotating the handle. A side port connected to the handle allows for contrast injections through the device, a useful feature that enables intermittent venography at the treatment site without requirement to remove the device. The device does not provide a mechanism for aspiration.

The device was delivered over an 0.35 superstiff Amplatz wire with through-and-through access in all but one case (owing to an inability to obtain dual access). The initial diameter of the coring element was chosen based on angiographic appearances of the occluded segment. The diameter was altered as the procedure progressed based on tactile feedback while advancing/retracting the device and imaging appearances of the diseased segment.

We used two main techniques while using the device. The first of these was a scrubbing technique, where the device was advanced forward and backward through the diseased vessel while simultaneously rotating the coring element in both clockwise and anticlockwise directions. We intermittently used the grasping mechanism while doing this in an attempt to remove segments of thrombus. The second technique used was a more deliberate stepwise advancement of the device through the diseased segment. Using this technique, the device was positioned at an area that required focused treatment. The coring element was then rotated approximately two turns in a clockwise direction and subsequently in an anticlockwise direction. Finally, an attempt was made to grasp any disrupted webs or thrombus.

Adjunctive use of the ClotTriever thrombectomy catheter was made in a number of cases. The ClotTriever device has a different mechanism of action to the VenaCore, more specifically designed for the removal of large volumes of acute thrombus from veins. The device is also delivered over a wire; however, it is initially advanced beyond the point of the thrombosis. The device, which consists of a nonrotational coring element and nitinol mesh collection basket, is then withdrawn back through the diseased segment. The coring element is designed to separate thrombus from the vessel wall and the thrombus then collects in the basket before being removed from the patient. Our decision to implement the ClotTriever catheter was made based on the venograms that were obtained after use of the VenaCore and subsequent balloon angioplasty. In a number of situations, where some residual irregularity/stenosis within the vessel had proven resistant to both VenaCore and balloon angioplasty, a number of passes with the ClotTriever were made in an attempt to maximize luminal gain and thrombus removal.

Final venography was performed to assess for caliber improvement and any immediate complications. Post procedure, the sheaths were removed and haemostasias was achieved with either manual compression or purse string suture. Patients were transferred to an observation unit where they were monitored for 3 hours before discharge.

### Anticoagulation

All patients treated were on anticoagulation therapy in the form of either rivaroxaban or apixaban. Given the importance of ensuring adequate anticoagulation in this group around the time of the procedure, while also being able to control our ability to discontinue anticoagulation in the case of procedural complication, all patients were transitioned to a therapeutic dose of low molecular weight heparin (tinzaparin) two days before the procedure. This was then continued for a duration of 6 weeks post procedure.

Intraprocedural anticoagulation was administered in the form of unfractionated heparin. The activated clotting time was monitored during the procedure. Given the pathophysiology of chronic venous occlusion, whereby thrombus has essentially resulted in scarring and fibrosis of the vein wall, tissue plasminogen activator is unlikely to be of benefit in these procedures and was not administered.

## Results

### Patient demographics

Eight consecutive patients who underwent percutaneous treatment for symptoms of PTS were treated with the VenaCore device between September 2024 and December 2024 (Table II). Four male and four female patients with a median age of 39 years were treated. The median time interval since initial diagnosis with DVT was 2 years; however, a broad range of time intervals were treated ranging from 6 months to >20 years. In the four male patients, the thromboses were considered unprovoked. In the four female patients, two were diagnosed with DVT during pregnancy and the other two patients were on a combined oral contraceptive pill. Thirty-seven percent of patients (n = 3) had undergone previous endovascular treatment in the form of balloon angioplasty. All patients had preprocedural imaging to delineate the extent of venous disease; 63% of patients had an US examination, and all patients had either magnetic resonance venography or computed tomography venography. Regarding symptomatology, all patients were considered to have symptoms of PTS based on the Villalta scoring system ranging from 8 to 16 (median, 12.0; interquartile range [IQR], 11.0-14.5). Clinical Etiological Anatomical Pathophysiological scores ranged from 2 to 4b (Table II). One patient was deemed to have mild symptoms; however, imaging demonstrated a persistent occlusive focus in the femoral vein after previous endovascular therapy. Given that this patient expressed a preference to have this removed, the patient was accepted as a candidate for treatment with the VenaCore device. Four patients had iliofemoral disease, and the other four were treated for femoral-popliteal occlusions.

**Table II:** Clinical presentations

Patient	Age range	Sex	Time since DVT diagnosis	Imaging	Symptoms	Prior intervention	Villalta score before procedure	CEAP	Villalta score after procedure
1	60-70	Male	1 year	US/MRI	Right leg: constant pain, swelling, cramps-above and below knee. Mild pruritis.	IV TPA and balloon angioplasty	12	3	7
2	30-40	Male	3 years	US/MRI	Left leg: swelling, worsening significantly with activity. Pain and erythema in leg following 10 minutes in standing position.	None	13	3	9
3	30-40	Male	6 months	US/MRI	Left leg: pain, swelling and pressure/heaviness	None	11	3	6
4	30-40	Female	2 years	US/MRI	Left leg: mild swelling and pain	Balloon angioplasty	8	2	5
5	30-40	Female	10 years	MRI	Left leg: chronic swelling and pain with difficulty ambulating. Pain worsened over last 2 years.	IV TPA and balloon angioplasty	16	4a	Reoccluded
6	40-50	Female	1 year	US/CTV	Left leg: thigh pain and swelling. Swelling and pain significantly worse with prolonged standing, sitting and walking long distances.	None	12	3	5
7	50-60	Female	20 years	CTV	Left pelvic pain. Left lower limb swelling. Venous claudication	None	16	4b	N/a
8	40-50	Male	3 years	MRI	Right leg: pain and swelling.	None	11	3	6

*CEAP*, Clinical Etiological Anatomical Pathophysiological; *CTV*, computed tomography venography; *DVT*, deep vein thrombosis; *IV*, intravenous; *MRI*, magnetic resonance imaging; *TPA*, tissue plasminogen activator; *US*, ultrasound.

### Procedural details

The median length of occluded segment crossed was 20 cm (IQR, 6-30 cm) with a median crossing time of 22 minutes (IQR, 6-28 minutes). The occluded segments were successfully traversed in all patients. Balloon angioplasty was performed along the diseased vein both before and after using the VenaCore device in all patients. Total VenaCore device time was a median of 10 minutes (IQR, 5-12 minutes) and the overall procedure time varied significantly based on extent of venous disease; however, a median time of 110 minutes was observed (IQR, 70-180 minutes).

A single case of intraprocedural vessel rupture was encountered using the VenaCore device. This was encountered in the patient with the most long-standing disease (20 years). The rupture was of the left external iliac vein and was managed successfully using balloon tamponade with a 10-mm Mustang balloon. Balloon tamponade was performed for an initial period of 4 minutes and subsequently a further 3 minutes. A final angiogram demonstrated cessation of extravasation; however, the recanalized segment had reoccluded, likely owing to some extrinsic compression from a perivascular hematoma. The patient reported pain in the left side of the pelvis; however, they remained hemodynamically stable throughout.

In the seven recanalized patients, multiple small fragments of thrombus (range, 5-12) were removed ranging in size from 1 to 3 cm. These small fragments demonstrated a fibrotic/chronic appearance (white/yellow color, firm consistency) in keeping with patients' duration of symptoms. No long continuous segments of thrombus were removed.

Final venography demonstrated luminal gain in the seven patients who were successfully recanalized with a median gain of 75% (IQR, 70%-80%).

### Outcomes

Postprocedural follow-up was performed on all patients with a clinical consultation and US scan (range, 30-60 days; median, 37 days; IQR, 32-35 days). Of those patients who were successfully recanalized, 86% (n = 6) remained patent at US follow-up. In the patient who experienced re-, no change in symptoms was observed. Improvement in symptoms was reported by the other six patients (Table II), with a mean reduction in Villalta score of 4.8 (IQR, 4.0-5.0) observed.

## Case examples

**Fig 1:**
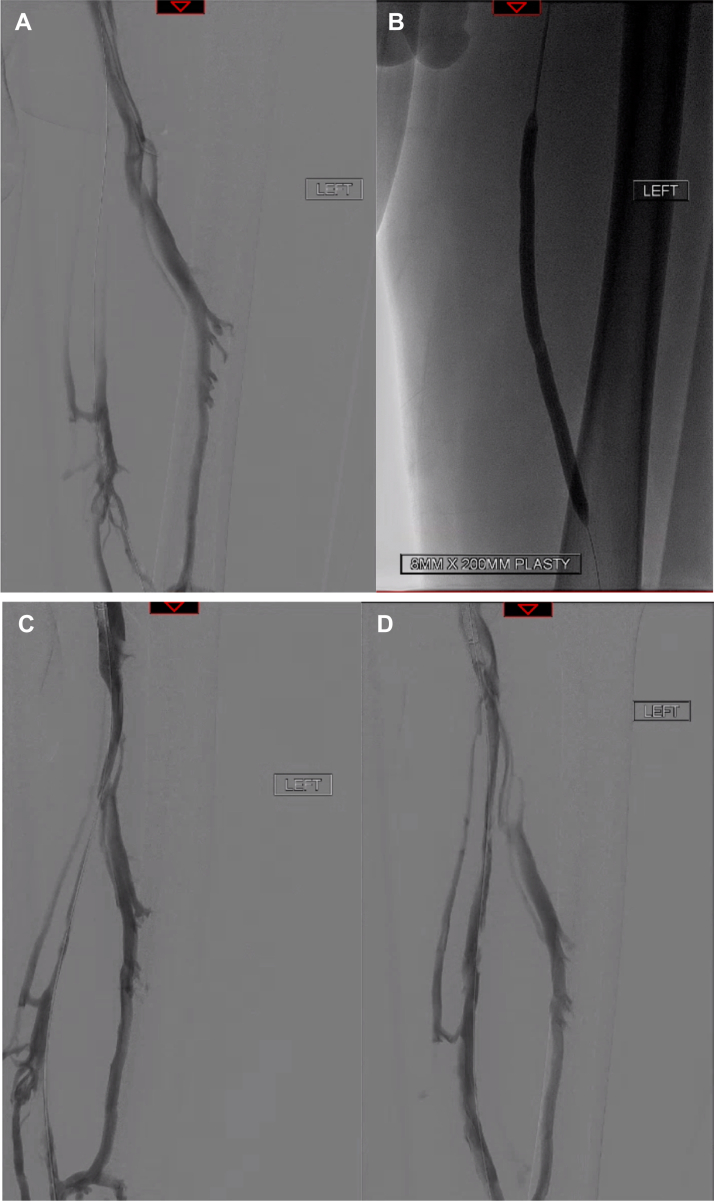
Occluded femoral vein with venous drainage predominantly through a patent profunda femoris vein **(A)**. Balloon angioplasty across the diseased segment was successful in creating a channel however significant elasticity resulted in persistent severe stenosis **(B and C)**. Following passage of the VenaCore device and repeat angioplasty, good luminal gain was achieved **(D)**.

**Fig 2:**
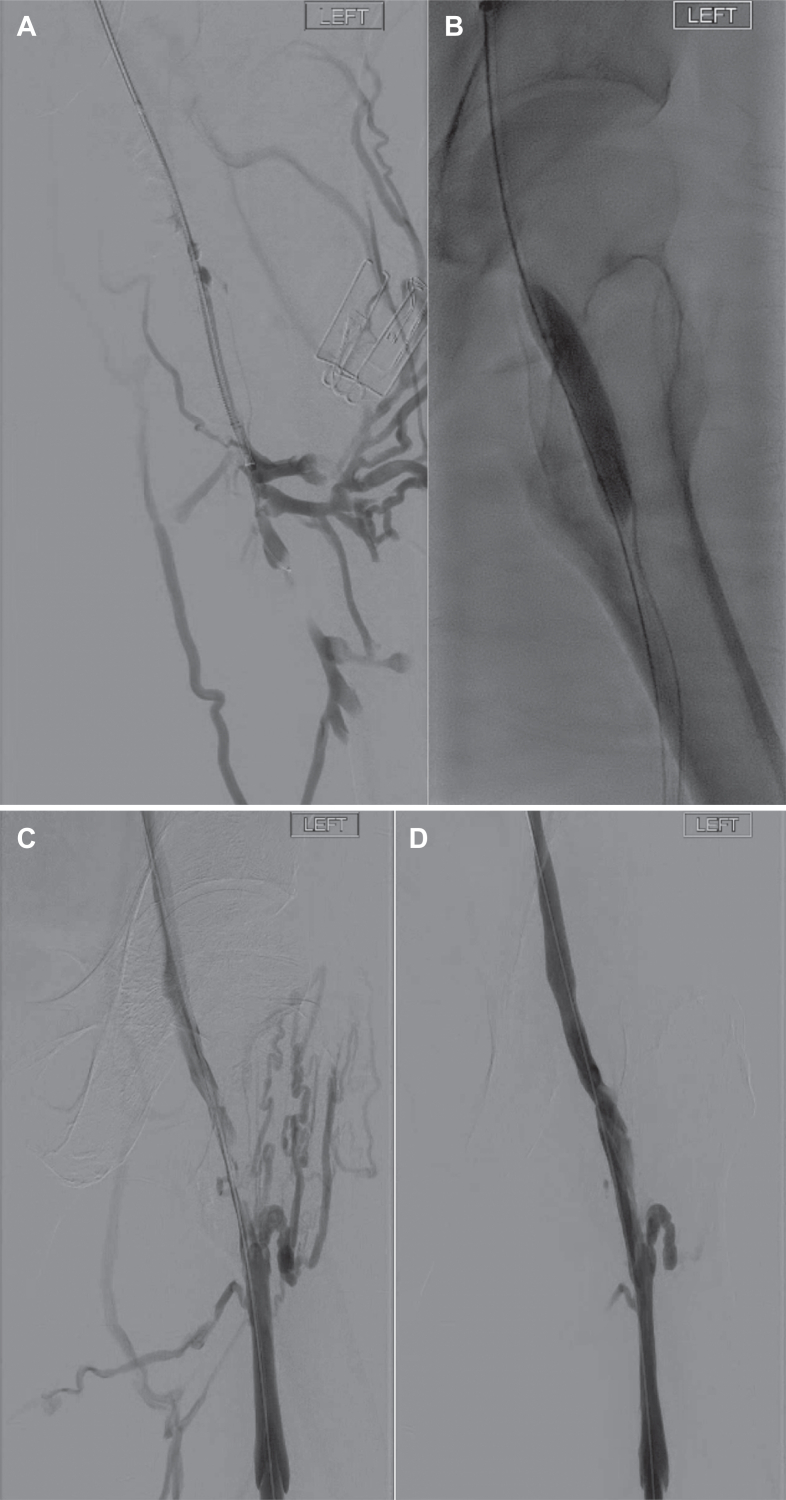
Short segment occlusion of the left femoral vein successfully crossed and angioplastied **(A and B)**. Postangioplasty imaging shows a persistent luminal irregularity with filling of venous collaterals **(C)**. Following use of the VenaCore catheter, marked improvement in luminal patency and reduction in collateral filling was achieved **(D)**.

**Fig 3:**
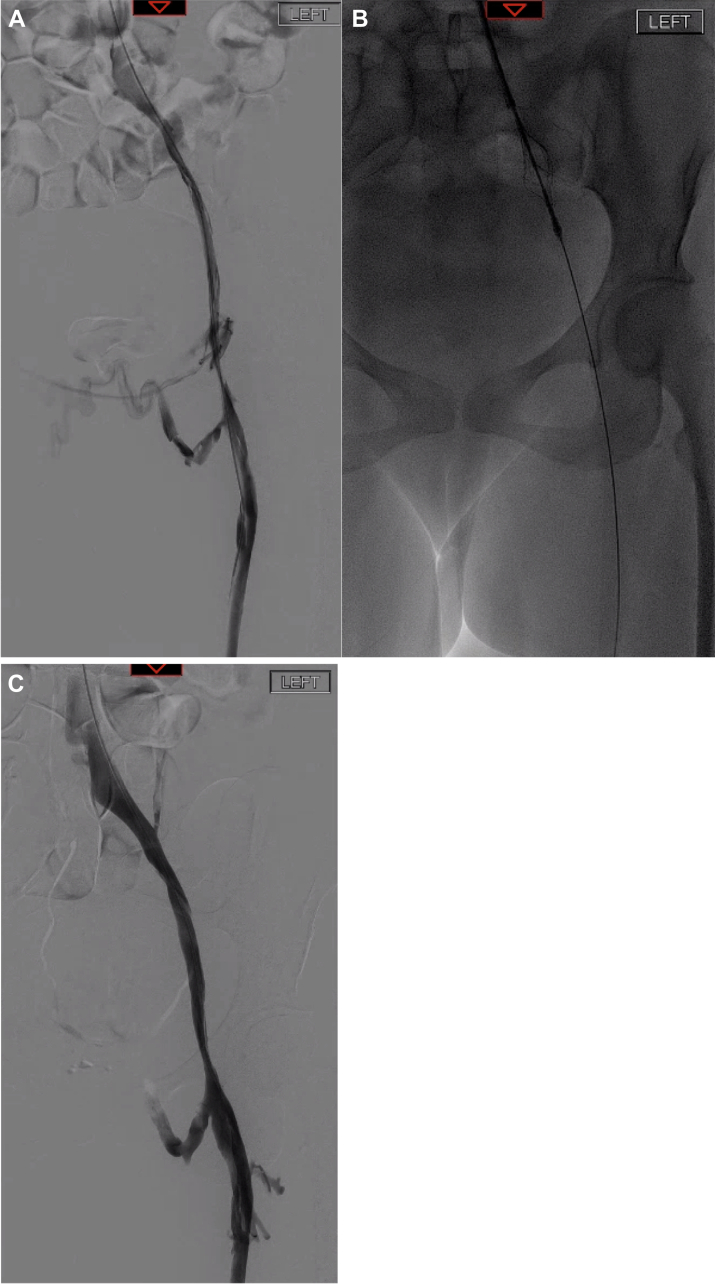
Following recanalization of the iliofemoral segment with balloon angioplasty, a channel was created through the diseased segment **(A)**. This was then treated using the VenaCore device and repeat angioplasty with marked improvement in luminal caliber of the diseased segment and reduction in cross pelvic collateral flow **(B and C)**.

**Fig 4:**
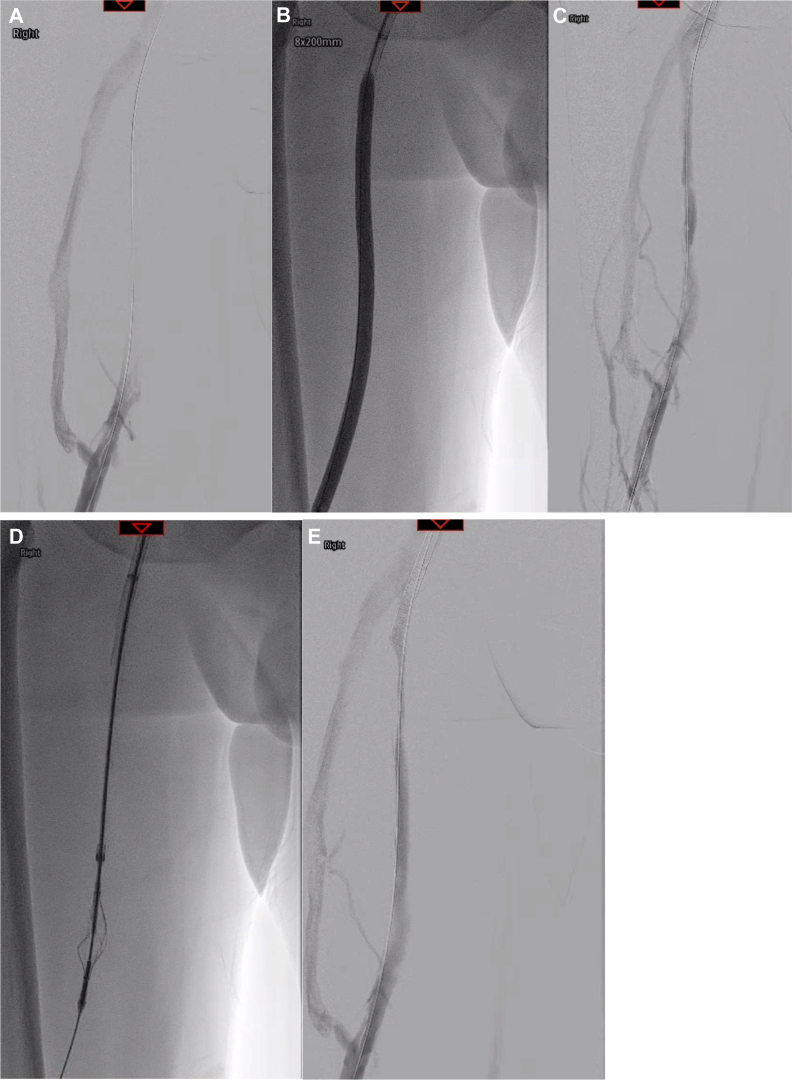
Occlusion of the femoral vein with collateral drainage via the profunda femoral vein **(A)**. After crossing of the lesion, balloon angioplasty was performed with creation of a channel however significant luminal irregularity was again noted **(B and C)**. Following passage of the VenaCore device, an immediate angiogram shows improved patency along the inferior and mid segments of the vein.

A number of case examples are provided below to demonstrate the pre and postprocedural angiographic appearances of the lesions treated with the VenaCore catheter (Fig 1, Fig 2, Fig 3, Fig 4).

## Discussion

We have outlined the technical and clinical results of our initial experience with the VenaCore thrombectomy catheter, demonstrating successful recanalization of the occluded segment in 88% of patients and patency rate of 86% at early follow-up. Our single case of intraprocedural vessel perforation occurred in a patient in whom the initial DVT was diagnosed 20 years previously. Our single case of early reocclusion was in a patient who was first diagnosed with DVT 10 years previously. These were the only patients in our cohort in whom symptoms were considered severe according to the Villalta scoring system and the outcomes underscore the significant challenges associated with treating those patients with long-standing chronic venous occlusion and severe PTS. Of the remaining six patients, those with both iliofemoral and femoral-popliteal segment disease were treated with the device and symptomatic improvement was noted in all 6 patients with mean reduction in Villalta score of 4.8 at early follow-up.

Although most of the data available surrounding percutaneous management of PTS relate to angioplasty and stenting, a previous study by Mouawad examined the role of percutaneous mechanical thrombectomy in the removal of post-thrombotic obstructions and subsequent impact on PTS associated venous leg ulceration (VLU).^12^ The authors reported on 11 patients with 15 VLUs who underwent thrombectomy. Of the 15 VLUs, 12 (80%) had resolved completely and 3 demonstrated near-complete healing. This study was performed before the release of the VenaCore device, which is designed specifically to address these challenging post-thrombotic obstructions. The authors in the study made use of the ClotTriever device, which has a broader range of applications. Within our cohort, we used the ClotTriever on four occasions in an attempt to improve upon areas of residual liminal narrowing after the use of the VenaCore and balloon angioplasty. We did not, however, identify any significant change on venography or remove any further thrombus using the ClotTriever after the VenaCore device.

In those patients with predominantly iliofemoral disease and otherwise patent deep venous system below the femoral vein, good venous return from the lower limb confers the possibility of iliac stent placement.^13^^,^^14^ A 2007 study by Neglén et al^15^ examined the outcomes of iliac stenting in a cohort of 464 post-thrombotic patients. The authors noted improvements in pain, swelling, and quality-of-life (QOL) parameters. More recently, a randomized control trial by Shekarchian et al^16^ examined impact on QOL after stenting for iliofemoral venous obstruction. Although the study reported issues relating to patient recruitment, the authors did note significantly higher VEINES-QOL/Sym scores at 12 months compared with a control group. In a 2022 study that examined the clinical efficacy of stenting in patients with PTS secondary to iliofemoral obstruction, Hoshino et al^17^ reported a reduction in median Villalta score from 16 to 7 in a cohort of 30 patients. An ongoing randomized controlled trial examining whether endovascular therapy is an effective strategy to reduce PTS disease severity and improve QOL in patients with established disabling iliac-obstructive PTS is approaching completion (C-TRACT trial).^18^ The study aims to recruit 250 subjects and will provide further insight into the potential impact of catheter directed therapy in treating this challenging condition. Although further research will be required to assess the potential efficacy of the VenaCore device as a lone therapy in the management of iliofemoral occlusions, we hypothesize that the device may act as a useful adjunct to stenting, particularly with regard to improving inflow.

There is increasing awareness of the important role that inflow plays in maintaining iliofemoral stent patency.^19^^,^^20^ Given the potential impact disease of the femoral vein can have on disrupting inflow, treatment of the femoral segment may be required.^21^ Within our cohort we noted luminal gain of between 70% and 80% after treatment of the diseased femoropopliteal segments. Where the VenaCore may, therefore, prove most useful in this group of patients is in those with iliofemoral disease which also extends some distance below the level of the inguinal ligament to involve the cranial aspect of the femoral-popliteal segment. The potential to address these chronic occlusions and improve flow through the upper thigh/common femoral vein, may allow for iliac stent placement in patients who otherwise would have been precluded owing to poor inflow.

Those patients with disease centered on the femoropopliteal segment present a different challenge. Data on therapeutic intervention for PTS secondary to femoropopliteal disease are scarce and the use of stents in this location remains controversial. A 2024 study by Zbinden et al^11^ reported on 29 patients who were treated with percutaneous transluminal angioplasty for PTS secondary to femoropopliteal disease. At a median follow-up of 395 days, primary patency of the femoropopliteal segments was 79.3%, with a secondary patency of 82.8%. The mean Villalta score decreased from 11.5 to 8.0.^11^ Further research with longer follow-up will be needed to ascertain whether combined therapy of VenaCore thrombectomy and percutaneous transluminal angioplasty can improve upon these results.

In many cases, an extensive network of collateral veins has developed around the occluded femoral-popliteal segment which can result in difficulty identifying and recanalizing the true lumen. IVUS can act as a useful tool in many of these cases. The utility of IVUS is well-documented for iliac vein stenting.^22^ In the group of patients undergoing thrombectomy, it also provides information regarding the severity of the occlusions and venous webs. It allows for an accurate assessment of luminal improvement after device use. Access to IVUS examination varies across countries and institutions. We used IVUS examination in our first case and it proved to be a useful adjunct for the reasons previously described. Subsequent cases were performed without IVUS examination owing to access constraints. Although accurate venography performed in multiple projections allows for these procedures to be performed in a safe manner, it does not provide the same level of detail on disease location and severity. We recommend the use of IVUS examination where available.

Despite the luminal gain achieved on post-VenaCore angiograms, we did not remove large segments (>5 cm) of clot in any of our cases. Given the pathophysiology of PTS, whereby diseased veins undergo remodeling with collagen deposition and scarring, in those patients with long-standing venous occlusion (>1 year), the likelihood of removing large volumes of thrombus is low.^23^ The devices main utility appears to be in improving luminal patency in conjunction with balloon angioplasty through its ability to core through the diseased segment, disrupting venous webs and other chronic venous synechiae.

## Conclusions

The VenaCore catheter is a novel tool aimed at addressing chronic venous occlusions. In our small cohort of patients with PTS, the VenaCore proved useful in improving luminal gain when used in conjunction with balloon angioplasty, with associated improvement in clinical symptoms. Although our early experience shows promise in recanalizing occluded venous segment and alleviating symptomatology of PTS with an acceptable safety profile, further research is required to assess the long-term patency rates and durability of symptom reduction.

## Author Contributions

Conception and design: AB

Analysis and interpretation: CR, AB

Data collection: CR, AB

Writing the article: CR

Critical revision of the article: AB

Final approval of the article: CR, AB

Statistical analysis: CR, AB

Obtained funding: Not applicable

Overall responsibility: AB

## Funding

None.

## Disclosures

A.B. reports consulting fees for sitting on advisory boards with Inari medical.
